# Knowledge, attitudes and practices (KAP) relating to avian influenza in urban and rural areas of China

**DOI:** 10.1186/1471-2334-10-34

**Published:** 2010-02-21

**Authors:** Nijuan Xiang, Ying Shi, Jiabing Wu, Shunxiang Zhang, Min Ye, Zhibin Peng, Lei Zhou, Hang Zhou, Qiaohong Liao, Yang Huai, Leilei Li, Zhangda Yu, Xiaowen Cheng, Weike Su, Xiaomin Wu, Hanwu Ma, Jianhua Lu, Jeffrey McFarland, Hongjie Yu

**Affiliations:** 1 Office for Disease Control and Emergency Response, Chinese Center for Disease Control and Prevention, Beijing, China; 2 Chinese Field Epidemiology Training Program, Chinese Center for Disease Control and Prevention, Beijing, China; 3 Anhui Provincial Center for Disease Control and Prevention, Hefei, China; 4 Shenzhen Center for Disease Control and Prevention, Shenzhen, China; 5 Chongqing Medical University, Chongqing, China; 6 Huanshan Center for Disease Control and Prevention, Huangshan, China; 7 Xiuning Center for Disease Control and Prevention, Xiuning, China; 8 Influenza Division, National Center for Immunization and Respiratory Diseases, Center for Disease Control and Prevention, Atlanta, USA

## Abstract

**Background:**

Studies have revealed that visiting poultry markets and direct contact with sick or dead poultry are significant risk factors for H5N1 infection, the practices of which could possibly be influenced by people's knowledge, attitudes and practices (KAPs) associated with avian influenza (AI). To determine the KAPs associated with AI among the Chinese general population, a cross-sectional survey was conducted in China.

**Methods:**

We used standardized, structured questionnaires distributed in both an urban area (Shenzhen, Guangdong Province; n = 1,826) and a rural area (Xiuning, Anhui Province; n = 2,572) using the probability proportional to size (PPS) sampling technique.

**Results:**

Approximately three-quarters of participants in both groups requested more information about AI. The preferred source of information for both groups was television. Almost three-quarters of all participants were aware of AI as an infectious disease; the urban group was more aware that it could be transmitted through poultry, that it could be prevented, and was more familiar with the relationship between AI and human infection. The villagers in Xiuning were more concerned than Shenzhen residents about human AI viral infection. Regarding preventative measures, a higher percentage of the urban group used soap for hand washing whereas the rural group preferred water only. Almost half of the participants in both groups had continued to eat poultry after being informed about the disease.

**Conclusions:**

Our study shows a high degree of awareness of human AI in both urban and rural populations, and could provide scientific support to assist the Chinese government in developing strategies and health-education campaigns to prevent AI infection among the general population.

## Background

As of July 1 2009, 436 confirmed human H5N1 avian influenza (AI) cases, resulting in 262 deaths (case fatality rate, 60.1%), have been reported since November 2003, most of them in southeast Asia [1]. There is general agreement on the potential spread to humans with devastating consequences [2]. Case-control studies during the 1997 outbreak in the Hong Kong Special Administrative Region (SAR) of China [3], Thailand [4], Vietnam [5] and China [6] during 2004-2005 revealed the visiting of poultry markets and direct contact with sick or dead poultry to be significant H5N1 risk factors.

In mainland China, 37 confirmed H5N1 cases have been reported since October 2005 [7]. The Chinese H5N1 cases comprised two different groups regarding patterns of exposure to poultry: 71.4% (10/14) of urban patients had visited wet poultry markets (live or freshly slaughtered poultry) before onset of the illness, whilst 81.8% (18/22) of rural patients had previously been exposed to backyard poultry and to sick or dead poultry [6,8].

To date, there are limited data on the knowledge, attitudes and practices (KAPs) associated with AI in the general population [9,10]. We conducted a two-stage, household-based cluster survey of KAPs regarding AI in an urban area (city of Shenzhen), where poultry markets are common, and in a rural area (Xiuning County), where backyard poultry is common. Focal points of the survey were: types of information commonly held, awareness of AI, emotional reactions to human AI, and practices associated with its prevention.

## Methods

### Study Sites

The study was performed in Shenzhen city; a typical large urban population located in Guangdong Province, southeast China, just across the border from Hong Kong SAR, and in Xiuning County (Anhui Province in the middle of China); a typical rural area with a population of 240,000.

### Sample Size

A two-stage probability proportional to size (PPS) sampling method was used. In stage one, we selected 30 primary sample units (PSUs) from the 639 communities in Shenzhen and 30 PSUs from the 259 villages in Xiuning, ranked by population using SAS (Survey Select Procedure with PPS-systematic sample method, version 9.0; SAS Inc, Cary, NC, USA). In stage two, 20 households in each community of Shenzhen and 30 households in each village in Xiuning were randomly selected. A sample size of 1,750 participants in Shenzhen was calculated to achieve a point estimation with an α-error of 0.05, based on an estimated prevalence (about 20%) of chickens purchased live in Hong Kong SAR [9]. For Xiuning, it was estimated that about 50% of households would raise backyard poultry, requiring a maximum sample of 2,700. In both cases, sample sizes took into account the different sampling methods and assumed a response rate of 80%.

### Survey Methodologies

Trained investigators from the Chinese Center for Disease Control and Prevention (China CDC, Beijing) and local CDCs described the purpose of the study to eligible participants or their proxies and obtained oral informed consent before the enrollment. In each household, every family member who met the inclusion criteria was administered a standardized, structured questionnaire (see Additional file 1; and Additional file 2) in the local language to assess KAPs of AI. The inclusion criteria were: proper communication skills, age ≥ 15 years, and residence in the specific investigational area for ≥ 3 months.

Most questions were closed-ended: participants were allowed to choose from a pre-existing set of answers (Yes/No/Unclear). Most variables derived from these questions were categorical, with the exception of age.

The level of knowledge of AI was assessed according to awareness of the issue, sources of information and demand for more information. We also asked both groups whether or not AI is an infectious condition (3 points), to describe its mode of transmission (2 points), whether or not it can be prevented (1 point), and to estimate the level of recovery after treatment (1 point). Only the urban group was asked whether infection is associated with hygiene in wet poultry markets (1 point), and only the rural group was asked about the relationship between AI and 'fowl plague' (Newcastle disease) (1 point). In order to be considered knowledgeable, a participant had to attain a score of five points or more out of eight, according to a Likert-type scale [11,14].

Concerns related to AI were assessed via two questions regarding concerns about family/friends and fear of visiting public places due to the risk of catching the virus. To each question a maximum of 3 points was assigned if the answer was 'yes'; 2 points were assigned to 'not sure'; 1 point was assigned to 'unknown'; and 0 points for 'no'. A person who scored 5 or 6 points was defined as concerned about AI.

Practices associated with preventing human AI were also assessed, with questions about hand washing and eating poultry after being informed about AI. Urban residents were asked about direct contact with live poultry when purchasing in a wet poultry market, including direct contact with cages of live poultry and subsequent inoculation of mucous membranes after touching poultry cages. Rural participants, more likely to have contact with backyard poultry, were questioned on their exposure to sick or dead poultry.

### Statistical Analysis

Data from the questionnaires were entered in duplicate and verified using EpiData software (Odense, Denmark; available at http://www.epidata.dk/). Data were analyzed with SPSS (version 13.0; SPSS Inc., Chicago, IL, USA). Median and range values were calculated for continuous variables, and were compared between urban and rural groups using the Wilcoxon rank-sum test. For categorical variables, frequencies for urban and rural groups were compared using the chi-square test. A backward variable selection method was used to select variables for multivariate analysis by univariate analysis. The multiple linear regression model was used to analyze possible influencing factors associated with participants' AI knowledge and attitudes.

### Study Approval

The study protocol was approved by the Institutional Review Board of the China CDC and was carried out in compliance with the Helsinki Declaration. The requirement for signed, informed consent was waived because no sensitive individual information or clinical specimens were collected from participants. Verbal informed consent was obtained from all participants.

## Results

4,414 (98%) of the 4,504 eligible participants agreed to participate in the study and completed the questionnaire. Fifteen participants in Shenzhen and one in Xiuning were excluded from the analysis due to incomplete responses. Residents in the Shenzhen group were significantly younger than those in Xiuning (*p *< 0.001), with a median age of 32 years and 46 years, respectively. The level of education (high school or above) was much higher among the urban residents compared to the rural villagers: 760 (42%) vs. 199 (8%), respectively (*p *< 0.001). A similar difference was observed in occupation levels: 244 (13%) of participants were homemakers in Shenzhen vs. 39 (2%) in Xiuning (*p *< 0.001).

The most common source of information about human AI was television, with 94% of urban residents and 92% of rural residents (*p *= 0.04) identifying television as the main source of information on AI. Urban Shenzhen residents were more likely than rural Xining residents to be informed through newspapers (45% vs. 7%, *p *< 0.001) or the internet (21% vs. 2%, *p *< 0.001). More residents in the Xiuning group obtained information through health professionals (26% vs. 7%, *p *< 0.001) and family/friends (55% vs. 29%, *p *< 0.001).

A significant percentage of participants in both groups requested more information (62% for the Shenzhen group and 76% for the Xiuning group, *p *< 0.001). However, approximately a quarter of the population in both groups did not care about this problem (Shenzhen 28% vs. Xiuning 22%, *p *< 0.001). Official announcements of a poultry outbreak were less frequently demanded, especially by the Xiuning group (*p *< 0.001). Television was the preferred source of further information in 92% of the Shenzhen group and 95% of the Xiuning group (*p *< 0.001) (Table 1).

**Table 1:** Sources of AI information among urban residents in Shenzhen and rural villagers in Xiuning, China

Variable	Residents in Shenzhen (n = 1,826) No. (%)	Villagers in Xiuning (n = 2,572) No. (%)	*P *value*
Had heard of AI	1,652 (90)	2,263 (88)	0.009
Information source^±^			
Television	1,518 (92)	2,118 (94)	0.041
Newspapers	740 (45)	158 (7)	< 0.001
Family/friends	477 (29)	1,235 (55)	< 0.001
Internet	339 (21)	47 (2)	< 0.001
Radio broadcast	194 (12)	149 (7)	< 0.001
Health facility	114 (7)	579 (26)	< 0.001
Requested more information on AI			
Yes	1,135 (62)	1,968 (76)	
No	182 (10)	47 (2)	< 0.001
Did not care	509 (28)	557 (22)	
Type of information requested on AI^±^			
Prevention	1,039 (92)	1,876 (95)	< 0.001
Basic knowledge	741 (65)	1,011 (51)	< 0.001
Therapy	629 (55)	851 (43)	< 0.001
Official announcement of poultry outbreak	476 (42)	181 (9)	< 0.001
Requested more information on AI through^±^			
Television*	1,044 (92)	1,922 (98)	< 0.001
Newspapers	546 (48)	228 (12)	< 0.001
Internet	276 (24)	68 (3)	< 0.001
Healthcare workers	154 (14)	798 (41)	< 0.001
Radio broadcast	147 (13)	203 (10)	0.025
Family/friends	135 (12)	777 (39)	< 0.001

*Frequencies between urban and rural groups were compared by chi-square test.

^± ^A multiple options question, participants can select more than one option.

Almost three-of all participants were aware AI is an infectious disease that can be prevented; 40% of participants in the Shenzhen group vs. 31% in the Xiuning group (*p *< 0.001) were aware the infection could be transmitted from poultry; however, only 4% of urban and 6% of rural participants were aware that humans could fail to recover fully with treatment. Overall, a greater proportion of urban participants were considered to be knowledgeable about human infection with AI (Shenzhen group 69% vs. Xiuning group 56%; *p *< 0.001) (Table 2). A comparison of knowledge scores associated with AI for all interviewed respondents in Shenzhen city and Xiuning county found the same result; that urban residents were more knowledgeable about AI than the rural villagers (*p *< 0.001) (Table 3).

**Table 2:** Knowledge associated with AI among residents in Shenzhen and rural villagers in Xiuning, China

Variables	Residents in Shenzhen (n = 1,826), No. (%)			Villagers in Xiuning (n = 2,572), No. (%)			*P *value*
		
	Yes	No	Unknown	Yes	No	Unknown	
Human AI is an infectious disease	1,352 (74)	149 (8)	325 (18)	1,903 (74)	52 (2)	617 (24)	< 0.001
Humans can be infected with AI virus from poultry	729 (40)	362 (20)	735 (40)	804 (31)	301 (12)	1,467 (57)	< 0.001
Human infection with AI virus can be prevented	1,432 (78)	44 (2)	350 (19)	1491 (58)	41 (2)	1,040 (40)	< 0.001
Humans may not fully recover from infection with AI virus after treatment	80 (4)	1,073 (59)	673 (37)	166 (6)	1,102(43)	1,304 (51)	< 0.001
Human infection with AI virus is associated with hygiene of wet poultry market	1,472 (81)	81 (4)	273 (15)	-	-	-	-
AI is not the same as fowl plague (Newcastle disease)	-	-	-	822 (32)	852 (33)	898 (35)	-
Knowledgeable about human AI infection	1,259 (69)	567 (31)	0 (0)	1,453 (56)	1,119 (44)	0 (0)	< 0.001

* Frequencies between urban and rural group were compared by chi-square test.

^† ^Scores on these questions are described in Materials and Methods.

**Table 3:** Knowledge scores associated with AI among urban residents in Shenzhen and rural villagers in Xiuning, China

Area	Median	Range	Inter Quartile Range (IQR)	Z*	*P *value
Shenzhen (N = 1,826)	6	0-8	4-7	-12.547	<0.001
Xiuning (N = 2,572)	5	0-8	3-6		

*. The Z statistic was obtained from the Wilcoxon rank-sum test for two independent samples.

Significant single factors for AI knowledge scores including gender, age, education level, occupation, and requests for AI information were incorporated as independent variables into a multivariate analysis. The results showed, for urban residents, age, education level, occupation, and requests for AI information were possible influencing factors of AI knowledge. For rural villagers, gender, age, education level, occupation, and requests for AI information were possible influencing factors of AI knowledge (Table 4).

**Table 4:** Multivariate analysis for possible influencing factors of knowledge scores among urban residents in Shenzhen and rural villagers in Xiuning, China

Residents in Shenzhen (N = 1,826)				
**Factors**	**Coefficients**	Std. Error*	**t**	***P *value**^**†**^

Constant	3.786	0.230	16.446	<0.001
Age group				
5-18^#^	-	-	-	-
19-39	0.211	0.113	1.860	0.063
40-59	-0.007		-0.002	0.331
≥60	0.015		0.013	0.590
Highest level of education				
None or kindergarten^#^	-	-	-	-
Primary school	0.711	0.251	2.828	0.005
Junior high school	1.529	0.232	6.597	<0.001
High school	1.945	0.242	8.027	<0.001
College or higher	2.499	0.252	9.919	<0.001
Occupation				
Employed^#^	-	-	-	-
Unemployed	-0.025		-0.029	0.974
Student	0.500	0.247	2.022	0.043
Homemaker	0.021		0.024	0.954
Retired	0.732	0.223	3.291	0.001
Requiring further information on avian influenza				
Yes^#^	-	-	-	-
No	-0.446	0.166	-2.681	0.007
Don't care	-1.979	0.114	-17.407	<0.001

Villagers in Xiuning (N = 2,572)				

Constant	5.003	0.172	29.150	<0.001
Females	-0.394	0.077	-5.143	<0.001
Age group				
5-18^#^	-	-	-	-
19-39	0.021		0.025	0.771
40-59	-0.029		-0.030	0.599
≥60	-0.796	0.099	-8.054	<0.001
Highest level of education				
None or kindergarten^#^	-	-	-	-
Primary school	0.479	0.105	4.570	<0.001
Junior high school	1.067	0.120	8.895	<0.001
High school	1.298	0.182	7.121	<0.001
College or higher	1.422	0.460	3.093	0.002
Occupation				
Employed^#^	-	-	-	-
Unemployed	-0.012		-0.015	0.913
Students	0.430	0.178	2.420	0.016
Homemakers	-0.012		-0.015	0.957
Retired	-0.016		-0.020	0.960
Requiring further information of avian influenza				
Yes^#^	-	-	-	-
No	-0.593	0.271	-2.191	0.029
Don't care	-2.819	0.098	-28.700	<0.001

^# ^Reference group.

* Std Errors for the non-significant items were not available by using SPSS software.

^† ^Obtained by multiple linear regression model.

Overall, the rural group was significantly more concerned *(p *< 0.001) than the urban group about human infection with AI virus, as measured by concern about AI infection of family and friends, fear of visiting public places and catching the virus, and general concern about human infection with the virus. A comparison of attitude scores associated with AI for all interviewed respondents in Shenzhen city and Xiuning county also found that rural villagers were more concerned than urban residents (*p *< 0.001) (Table 5).

**Table 5:** Attitude scores associated with AI among urban residents in Shenzhen and rural villagers in Xiuning, China

Area	Median	Range	IQR	Z*	*P *value
Shenzhen (N = 1,826)	3	0-6	0-5	-13.862	<0.001
Xiuning (N = 2,572)	3	0-6	2-6		

*. The Z statistic was obtained from the Wilcoxon rank-sum test for two independent samples.

Significant single factors for AI attitude scores including gender, age, education level, occupation, requests for AI information, and knowledge scores were incorporated as independent variables into a multivariate analysis. The result showed that, for urban residents, gender, age, education level, occupation, requests for AI information, and knowledge scores were possible influencing factors of AI concern. For rural villagers, gender, education level, requests for AI information, and knowledge scores were possible influencing factors of AI concern (Table 6).

**Table 6:** Multivariable analysis for possible influencing factors of attitude scores among urban residents in Shenzhen and rural villagers in Xiuning, China

Residents in Shenzhen (N = 1,826)				
**Risk factors**	**Coefficients**	Std. Error*	**t**	***P *value**^**†**^

Constant	2.224	0.216	10.275	<0.001
Female	0.263	0.099	2.658	0.008
Age group				
5-18^#^	-	-	-	-
19-39	-0.011		-0.011	0.934
40-59	0.017		0.016	0.831
≥60	-0.517	0.223	-2.319	0.020
Highest level of education				
None or kindergarten^#^	-	-	-	**-**
Primary school	0.479	0.155	3.087	0.002
Junior high school	0.022		0.010	0.178
High school	-0.281	0.122	-2.298	0.022
College or higher	-0.655	0.145	-4.531	<0.001
Occupation				
Employed^#^	-	-	-	-
Unemployed	0		0	0.988
Student	-0.552	0.243	-2.273	0.023
Homemaker	-0.031		-0.030	0.840
Retired	-0.017		-0.014	0.606
Requiring further information of avian influenza				
Yes^#^	-	-	-	-
No	-0.961	0.167	-5.753	<0.001
Don't care	-0.815	0.123	-6.634	<0.001
Knowledge score	0.144	0.023	6.194	<0.001

Villagers in Xiuning (N = 2,572)				

Constant	3.092	0.170	18.148	<0.001
Females	0.286	0.078	3.667	<0.001
Highest level of education				
None or kindergarten^#^	-	-	-	-
Primary school	0.014		0.015	0.987
Junior high school	-0.021		-0.022	0.895
High school	-0.022		-0.023	0.957
College or higher	-0.864	0.473	-1.826	0.068
Requiring further information of avian influenza				
Yes^#^	-	-	-	-
No	-1.563	0.287	-5.445	<0.001
Don't care	-1.282	0.117	-10.992	<0.001
Knowledge score	0.140	0.020	7.014	<0.001

^# ^Reference group.

* Std Errors for the non-significant items were not available by using SPSS software.

^† ^Obtained by multiple linear regression model.

Among the practices used to prevent human AI transmission, 1,152 (64%) of urban participants used soap for hand washing vs. 965 (38%) among the rural group (*p *< 0.001). Other practices (use of detergent for dish washing, anti-bacterial solutions, washing powder and shampoo) were similarly, but rarely, adopted by the two groups (7% to <1%). Regarding eating habits, 45% (714/1,601) of Shenzhen participants reported eating less poultry since becoming aware of this infectious disease, whereas 33% (732/2,213) of participants in the Xiuning group reported eating less poultry (*p *< 0.001).

The majority of urban residents (1,693, 93%) never had direct contact with poultry or their cages when purchasing them. A significant majority of those who had contact with either poultry or cages never subsequently touched their mouth, nose, or eyes (92/112, 82%). Similarly, most rural residents stated they infrequently had direct contact with sick or dead poultry (2,409, 95%). The majority of those who had direct contact with sick or dead poultry washed their hands after contact (119/133, 89%).

## Discussion

Despite certain limitations in the study methodology, mainly due to the need to produce simple questions understandable to all groups, our study revealed a number of important differences between KAPs of urban and rural populations for AI. Multivariate analysis demonstrated that age and level of education were likely the main factors giving rise to these differences. The study showed a high degree of awareness of human AI in both urban and rural regions. Awareness evaluations in Thailand [12] and Cambodia [13] after their 2004-2005 AI outbreaks gave similarly high percentages.

Television was found to be the most effective way to disseminate information on AI in both groups, followed by newspapers in the urban group and family/friends in the rural community. A significant percentage of both groups requested additional information; television, again, being the preferred source. These results confirm findings in Thailand where television proved to be the most efficient source of information [12].

The percentage of participants with 'knowledge associated with human AI' was low, especially in the rural areas where education levels were lower. Olsen et al [12] also found very low percentages of basic knowledge of human AI in rural Thailand before public education campaigns. However, in Italy, Abbate et al [11] found 64% of 284 poultry workers correctly defined AI as a contagious infection caused by a virus that can affect all species of birds. Nearly all workers identified poultry and wild birds as common vectors. These percentages are much higher than our results here. A recently published Italian study showed that knowledge of AI was greater among more highly educated populations, with a similar percentage aware that AI was preventable [14].

The urban group in this survey was less concerned about human infection with AI than their rural counterparts, probably due to their greater awareness of AI, in part, resulting from their higher level of education. In the Hong Kong SAR, with its high level of education, a telephone survey found 36% of respondents agreed that buying live chickens was a health risk and only 9% estimated a >50% likelihood of resultant sickness [9]. In addition, a telephone survey conducted in Hong Kong SAR in 2005 [15], which evaluated perceptions related to human AI and its association with anticipated psychological and behavioral responses in an outbreak, revealed that only 19.9% of participants avoiding visiting hospitals due to fear of AI, and a general education level (42.4% was matriculated or above) higher than in our study population.

Multivariate analysis results suggested, for both populations in our study, a possible relationship between AI knowledge, some of the demographic characteristics, and requests for AI information existed, as well as a possible relationship between AI attitude, some of the demographic characteristics, knowledge scores, and requests for AI information. However, a quantitative relationship cannot be evaluated from the data in our present study, and such an issue could be explored by further investigations in the future.

Our study showed a higher level of proper hygienic practice among urban residents. A study by Olsen et al. in Thailand before the 2004 outbreak demonstrated similar results among rural residents [12]. Another study conducted in Italy among the general population showed low compliance with precautionary behavior [14]. All implied the practice of precautionary activities in avoiding infection by AI virus needs to be strengthened.

Both groups in the present study reported a higher proportion of eating habit changes after awareness of the spread of AI, especially the rural villagers, who had more concern about AI, comparison with a survey conducted by Lau et al [15]. Facing AI, a new, emerging infectious disease with a high fatality rate, it is expected that people will have varying degrees of concern, ranging from indifference to panic. Timely and comprehensive public-risk communications from the government or other professional agencies are necessary to appease the possible negative social psychological influences such an outbreak would bring, in addition to the importance of persuading the public to take appropriate attitudes towards their practices of disease control and prevention.

Despite the novelty and significance of these findings, one methodological consideration that ought to be highlighted when interpreting these results is that the scores of AI knowledge and attitudes were not validated in China.

## Conclusions

Studies conducted in China, Thailand and Vietnam revealed that risk factors for H5N1 infection included recent exposure to live poultry, direct contact with dead poultry that had died of unknown causes [8], and the presence in the household of dead or sick poultry [3-6,9,10,13]. This study investigated the levels of knowledge, attitude and practices regarding these risk factors and could provide scientific support to assist the Chinese government in developing strategies and health education campaigns to prevent transmission of the AI virus among the general population. These campaigns should include such advice as avoidance of direct contact with sick or dead poultry, and use of protective equipment such as gloves and masks when contact is unavoidable. Such campaigns should utilize television as the primary medium of dissemination in all localities, with newspapers and the internet being the secondary source in urban areas, and local opinion leaders, such as family/friends and local doctors, taking a significant role in rural areas.

## Competing interests

The authors declare that they have no competing interests.

## Authors' contributions

HY contributed to study design; NX, YS, JW, and SZ participated in the field investigations, collected data from participants for the study, and helped to analyze the data; HY drafted the manuscript; JM helped to revise the manuscript. All other co-authors participated in collection and management of data. All authors read and approved the final manuscript.

## Pre-publication history

The pre-publication history for this paper can be accessed here:

http://www.biomedcentral.com/1471-2334/10/34/prepub

## Supplementary Material

Additional file 1 **Questionnaire S1 Questionnaire S1 Questionnaire of population based assessment of avian exposure and knowledge, attitudes and practices (KAPs) associated with avian influenza in Shenzhen**. This structured questionnaire had been used in our study to collect information about knowledge, attitudes and practices (KAPs) associated with avian influenza in Shenzhen. Click here for file

Additional file 2 **Questionnaire S2 Questionnaire of population based assessment of avian exposure and knowledge, attitudes and practices (KAPs) associated with avian influenza in Xiuning**. This structured questionnaire had been used in our study to collect information about knowledge, attitudes and practices (KAPs) associated with avian influenza in Xiuning. Click here for file

## References

[B1] World Health Organization Cumulative number of confirmed human cases of avian influenza A/(H5N1) reported to WHO 2009 http://www.who.int/csr/disease/avian_influenza/country/cases_table_2009_07_01/en/index.html Accessed July 3

[B2] Writing Committee of the Second World Health Organization Consultation on Clinical Aspects of Human Infection with Avian Influenza A (H5N1) virus. Update on Avian Influenza A (H5N1) Virus Infection in Humans N Engl J Med 2008 358 261 273 10.1056/NEJMra0707279 18199865

[B3] Mounts AW Kwong H Izurieta HS Ho Y-y Au TK Lee M Bridges CB Williams SW Mark KH Katz JM Thompson WW Cox NJ Fukuda K Case-control study of risk factors for avian influenza A (H5N1) disease, Hong Kong, 1997 J Infect Dis 1999 80 505 508 10.1086/314903 10395870

[B4] Areechokchai D Jiraphongsa C Laosiritaworn Y Hanshaoworakul W O'Reilly M Investigation of avian influenza (H5N1) outbreak in humans - Thailand, 2004 Morb Mortal Wkly Rep 2006 55 S3 S6 16645574

[B5] Dinh PN Long HT Tien NTK Hien NT Mai LTQ Phong LH Tuan LV Tan HV Nguyen NB Tu PV Phuong NTM WHO/Global Outbreak Alert and Response Network Avian Influenza Investigation Team in Vietnam Risk factors for human infection with avian influenza A H5N1, Vietnam, 2004 Emerg Infect Dis 2006 12 1841 1847 17326934 10.3201/eid1212.060829 PMC3291373

[B6] Zhou L Liao Q Dong L Huai Y Bai T Xiang NJ Shu YL Liu W Wang SW Qin PZ Wang M Lv J Chen RY Feng ZJ Yang WZ Uyeki TM Yu Hongjie Risk factors for human illness with avian influenza A (H5N1) virus infection in China, 2005-2007 J Infect Dis 2009 199 1726 1734 10.1086/599206 19416076 PMC2759027

[B7] Yu HG Shu YL Hu SX Zhang H Gao ZC Chen HL Dong J Xu CL Zhang Y Xiang NJ Wang M Guo YJ Cox NJ Lim W Wang Y Yang WZ The first confirmed human case of avian influenza A (H5N1) in mainland China Lancet 2006 367 84 16875527 10.1016/S0140-6736(05)67894-4

[B8] Yu HG Feng ZJ Zhang XF Xiang NJ Huai Y Zhou L Li ZJ Xu CL Luo HM He JF Guan XH Yuan ZA Li YT Xu LS Hong RT Liu XC Zhou XY Yin WW Zhang SX Shu YL Wang MW Wang Y Lee CK Uyeki TM Yang WZ the Avian Influenza H5N1 Study Group Human influenza A (H5N1) cases, urban areas of People's Republic of China, 2005-2006 Emerg Infect Dis 2007 13 1061 1064 18214180 10.3201/eid1307.061557 PMC2878233

[B9] Fielding R Lam WWT Ho EYY Lam TH Hedley AJ Leung GM Avian influenza risk perception, Hong Kong Emerg Infect Dis 2005 11 677 682 15890118 10.3201/eid1105.041225 PMC3320362

[B10] Fielding R Bich TH Quang LN Lam WWT Leung GM Tien TQ Ho EYY Anh LV Live poultry exposures, Hong Kong and Hanoi, 2006 Emerg Infect Dis 2007 13 1065 1067 18214181 10.3201/eid1307.061031 PMC2878218

[B11] Abbate R Di Giuseppe G Marinelli P Angelillo IF Knowledge, attitudes and practices of avian influenza, poultry workers, Italy Emerg Infect Dis 2006 12 1762 1765 17283632 10.3201/eid1211.060671 PMC3372355

[B12] Olsen SJ Laosiritaworn Y Pattanasin S Prapasiri P Dowell SF Poultry-handling practices during avian influenza outbreak, Thailand Emerg Infect Dis 2005 11 1601 1603 16318704 10.3201/eid1110.041267 PMC3366731

[B13] Ly S Van Kerkhove MD Holl D Froehlich Y Vong S Interaction between humans and poultry, rural Cambodia Emerg Infect Dis 2007 13 130 132 10.3201/eid1301.061014 17370527 PMC2725837

[B14] Di Giuseppe G Abbate R Albano L Marinelli P Angelillo IF A survey of knowledge, attitudes and practices towards avian influenza in an adult population of Italy BMC Infect Dis 2008 8 36 44 10.1186/1471-2334-8-36 18366644 PMC2292195

[B15] Lau JTS Kim JH Tsui H Griffiths S Perceptions related to human avian influenza and their associations with anticipated psychological and behavioral responses at the onset of outbreak in the Hong Kong Chinese general population Am J Infect Control 2007 35 38 49 10.1016/j.ajic.2006.07.010 17276790 PMC7132695

