# Supplementary material for: Knowledge, attitudes and practices (KAP) relating to avian influenza in urban and rural areas of China
Source: BMC Infect Dis. 2010 Feb 21;10:34. doi: 10.1186/1471-2334-10-34 (PMC2838887; doi:10.1186/1471-2334-10-34)
Supplement: Additional file 2 — Questionnaire S2 Questionnaire of population based assessment of avian exposure and knowledge, attitudes and practices (KAPs) associated with avian influenza in Xiuning. This structured questionnaire had been used in our study to collect information about knowledge, attitudes and practices (KAPs) associated with avian influenza in Xiuning. [file 1471-2334-10-34-S2.PDF]

Additional file 2, Questionnaire S2
Title: Questionnaire of population based assessment of avian exposure and knowledge, attitudes and practices (KAPs) associated with avian influenza in Xiuning
Description: This structured questionnaire had been used in our study to collect information about knowledge, attitudes and practices (KAPs) associated with avian influenza in Xiuning

Questionnaire of Population Based Assessment of Avian Exposure and Knowledge, Attitudes and Practices (KAPs) Associated with Avian Influenza in Xiuning

Good morning/afternoon/evening.

I am from China CDC. This investigation is a population based assessment of avian exposure and knowledge, attitudes and practices (KAPs) associated with avian influenza in the Shenzhen and Xiuning area of China, and has been organized by China CDC. From this study we can get the prevalence of exposure to sick/dead poultry and the prevalence of KAPs associated with AI that could help the government develop strategies and health education campaigns in AI control and prevention.

You have been selected randomly from Xiuning county; we would like to know some information about your exposure to sick/dead poultry, raising poultry, and knowledge, attitudes and practices relating to bird flu. No personal information will be gathered. I am going to ask you several questions. Please understand that there is no right or wrong answer; just answer the questions to the best of your ability. We need you to answer all questions by yourself (For children: if you can't understand the questions you can get help from your parents or guardian). It will take about 20-30 minutes to help me answer these questions. If you would like to take part in this survey we promise that all the answers will only be used by the researchers and you will get a gift after you finish the questionnaire. You can feel free to stop the survey at any time if you like, and if you have any questions please tell me and I'll try my best to help you.

If you consent to complete the questionnaire, let's begin. Thank you!

Consent by subject: □
Consent by parents or guardian: □

Date:___________Year___________Month__________Day

Chinese Center for Disease Control and Prevention
2007-6

Household ID:________________
Family number ID:___________________

Part 1: Demographic information
1. Gender:
① Male
② Female
2. How old are you?______ Or birth date: _____/_____/_____/(y/m/d)
3. What is your highest education level?
① Illiterate
② Primary school
③ Middle school
④ High school
⑤ College and above
4. What is your occupation?
① Farmer
② other___________

Part 2: Exposure to sick/dead poultry in backyard
1. Have you had direct contact with dead poultry in the past year? (If answer ② OR ③, then go to PART 3)
①Yes
②No
③Unclear
2. Did you wash your hands after touching the dead poultry?
①Yes
②No
③Unclear

Part 3: KAPs of avian influenza (<15 years needn't answer this part)
1. Have you heard about bird flu?
①Yes
②No (If answer ②, then go to QUESTION 15)
2. From where did you learn about the bird flu?
① Television
② Radio
③Newspaper
④Internet
⑤Friends/family
⑥Health care facility

⑦Other______
3. Is bird flu the same as avian plague?
①Yes
②No
③Unclear
4. Is bird flu a kind of infectious disease?
①Yes
②No
③Unclear
5. Can people get bird flu by touching sick poultry?
①Yes
②No
③Not sure
④Unclear
6. Can bird flu be prevented?
①Yes
②No
③Not sure
④Unclear
7. Can bird flu be cured?
①Yes
②No
③Not sure
④Unclear
8. Do you worry about your family/ friends getting bird flu?
①Yes
②No
③Not sure
④Unclear
9. Do you fear going to public areas due to bird flu?
①Yes
②No
③Not sure
④Unclear
10. Do you believe that the government can control the bird flu pandemic?
①Yes
②No
③Not sure
④Unclear
11. Would you like to get more information about bird flu? (If answer ② OR ③, then go to QUESTION 14)
①Yes
②No
③Don't care

12. What special information on bird flu would you like to know?
①Basic knowledge
②Prevention methods
③Treatment
④Epidemic announcements
⑤Other_____
13. What are the best ways to get this information to you?
① Television
② Radio
③ Newspaper
④ Internet
⑤Friends/family
⑥Health care facility
⑦Other______
14. Compared to the period of time before the spread of bird flu, would you say that you are eating:
①More poultry
②Less poultry
③Almost the same amount of poultry
④No poultry
⑤I never eat poultry
15. How do you usually wash your hands?
①With water
②With water and soap
③With an antibacterial solution
④Other________
⑤Never wash hands

The interview is finished, thank for your cooperation!

Interviewer:_________________ Interview date: _____/_____/_____/(y/m/d)
Check:____________________ Check date: _____/_____/_____/(y/m/d)
